# Safety evaluation of the antimicrobial peptide bovicin HC5 orally administered to a murine model

**DOI:** 10.1186/1471-2180-13-69

**Published:** 2013-03-27

**Authors:** Aline Dias Paiva, Kenner Morais Fernandes, Roberto Sousa Dias, Alípio dos Santos Rocha, Leandro Licursi de Oliveira, Clóvis Andrade Neves, Sérgio Oliveira de Paula, Hilário Cuquetto Mantovani

**Affiliations:** 1 Departamento de Microbiologia, Universidade Federal de Viçosa, Viçosa, Minas Gerais, Brazil; 2 Departamento de Biologia Geral, Universidade Federal de Viçosa, Viçosa, Minas Gerais, Brazil

**Keywords:** Bacteriocin, Lantibiotic, *Streptococcus bovis* HC5, BALB/c mice, Ovalbumin

## Abstract

**Background:**

Bovicin HC5 is an antimicrobial peptide that shows a broad spectrum of activity and potential for biotechnological and therapeutic applications. To gain insight about the safety of bovicin HC5 application, the histological and immunostimulatory effects of orally administrated bovicin HC5 to BALB/c mice were evaluated. BALB/c mice were divided into three groups: negative control (NC group); mice given purified bovicin HC5 (Bov group); mice given ovalbumin (positive control, PC group; a murine model of enteropathy). The mice were initially pre-sensitized, and PBS, bovicin HC5 or ovalbumin were administered for 30 days by daily gavages. Histological and morphometric analysis were performed and the relative expression of cytokines was analyzed by real-time RT-PCR.

**Results:**

The oral administration of bovicin HC5 to BALB/c mice reduced weight gain and caused alterations in the small intestine, although absorptive changes have not been detected. The number of total goblet cells and the mucopolysaccharides production were not affected by bovicin HC5 administration. A hypertrophy of Paneth cells and an increase in the number of mitotic cells were observed in Bov group, while the number of mast cells remained unaltered. Increased expression of TNF-α, INF-γ and IL-12 was observed in the small intestine upon bovicin HC5 administration.

**Conclusion:**

Bovicin HC5 has only minor effects on intestinal permeability and did not elicit an allergenic response upon oral administration to animal models. Considering the low *in vivo* toxicity of bovicin HC5, it might be a good candidate for enteral applications.

## Background

Bacteriocins are antimicrobial peptides produced by many species of bacteria and some members of the Archaea domain. Nisin, the most well-known bacteriocin, is produced by *Lactococcus lactis* strains and it belongs to the lantibiotic class of bacteriocins; nisin has GRAS status (Generally Recognized as Safe) and is currently the only bacteriocin approved for use as a food preservative [1]. Other bacteriocins, such as pediocin PA-1/AcH and lacticin 3147, are also commercially available, but are marketed as fermentates of lactic acid bacteria (LAB) having GRAS status [2].

The targeted mechanism of action and the relatively low propensity to select resistant bacteria are attractive properties of the lantibiotics. Moreover, previous studies have demonstrated the efficacy of many lantibiotics against target bacteria [3] and also the potential for biotechnological and therapeutic applications of these peptides [4]. Despite the good results obtained *in vitro*, the large scale application of lantibiotics remains limited due to the lack of data regarding clinical aspects, including the destiny of the peptides after ingestion, the loss of antimicrobial activity, the cytotoxicity and the immunostimulatory effects triggered by these peptides *in vivo *[5].

In order to evaluate the *in vivo* toxicity, an antimicrobial peptide should be administered daily and repeatedly to an animal model for a required period of time [6,7], and the route of administration should be the same proposed for use *in vivo *[8]. Because lantibiotics generally have low molecular mass and little intrinsic immunogenicity, coupling of these peptides to protein carriers or the use of adjuvants can be useful strategies to enhance the immunogenicity [9,10].

Bovicin HC5, a lantibiotic produced by the ruminal bacterium *Streptococcus bovis* HC5, has desirable properties, such as broad spectrum of activity, stability to low pH and high temperatures [11,12]. The mechanism of action of bovicin HC5 was recently elucidated and it is based on the specific interaction with lipid II molecule, leading to inhibition of the bacterial cell wall synthesis and eventually to pore-formation [13]. Previous results indicated that the *in vitro* toxicity of bovicin HC5 against mammalian cells is comparable to nisin [14]. Bovicin HC5 has been suggested as a potential alternative to classical antibiotics in livestock production and as an additive for food preservation [15,16].

To gain insight about the safety use of bovicin HC5 on animal hosts, we analyzed the effects of orally administrated bovicin HC5 to BALB/c mice, focusing on gastrointestinal permeability, morphological alterations in the GI tract and the immunostimulatory effects of the peptide. We used a murine model of enteropathy induced by sensitization to compare the effects caused by the administration of bovicin HC5.

## Results

### The administration of bovicin HC5 induces less weight gain in BALB/c mice

The weight of BALB/c mice was monitored during the trial period to verify if the sensitization followed by challenge with bovicin HC5 or ovalbumin affected weight gain of the animals, which could indicate clinical manifestation of allergy or gastrointestinal disorders. Symptoms as diarrhea, intestinal bleeding or rectal prolapsed were not observed.

Prior to the experiment, no significant differences were detected among the average weight of the mice (18.5, 18.4 and 18.3 g to NC, Bov and PC groups, respectively). In the NC group, the average mice weight ranged from 18.5 ± 0.35 g (day 0) to 20.8 ± 0.31 g (day 58), or a weight gain of 11.01% along the trial period.

**Figure 1:**
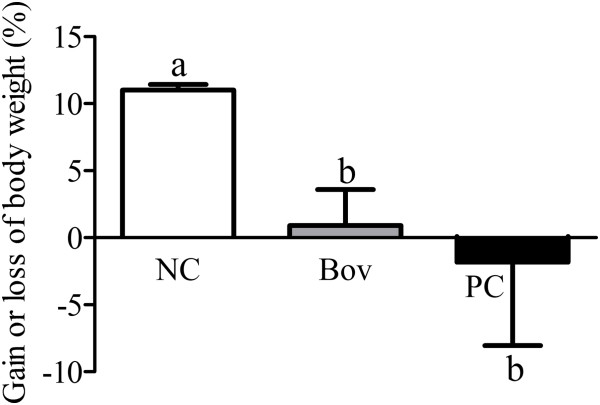
**Gain or loss of body weight in BALB/c mice during the experimental period.** The gain/loss of weight is shown as percentage of the animals’ weight, which was calculated comparing the weight at the end of the experiment (day 58) to the weight at the day of the first immunization (day 0). Each bar represents the percentage of weight gain obtained from two independent experiments, with the standard deviation (SD) (N = 8 animals per group). Statistically significant differences among treatments by the Dunn’s multiple comparison test (p < 0.05) were indicated by different lowercase letters (“a” or “b”) above the error bars. (NC) negative control group; (Bov) mice treated with bovicin HC5; (PC) positive control group.

Animals sensitized with bovicin HC5 or ovalbumin gained weight only during the three initial weeks of the experiment, before starting the oral administration of bovicin HC5 or ovalbumin. After 58 days of experiment, the percentage of weight gain was 0.91 and −1.8% for animals of the Bov and PC groups, respectively, which was significantly lower compared to the NC group (p < 0.05). There was no significant difference of weight gain between the Bov and PC groups (Figure 1).

### Gastrointestinal permeability is not altered upon oral administration of bovicin HC5

**Figure 2:**
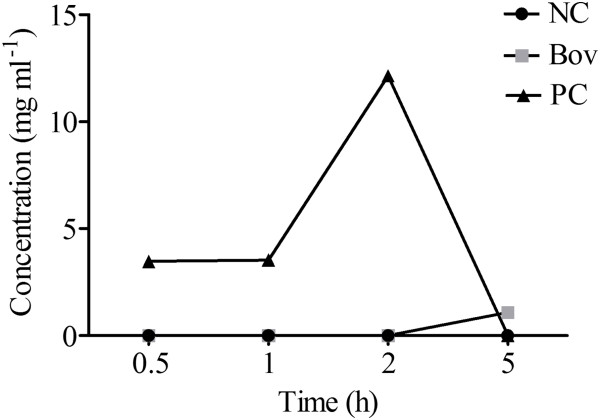
**Concentration of β-lactoglobulin in animal sera from treatment groups.** Upon an intragastrically dose of β-LG, blood was collected at the indicated time points and the levels of β-LG in mice sera were determined by FPLC. The results are shown as the average of β-LG concentration detected in a pool of animal’s sera from each experimental group (N = 8 mice per group), in two independent experiments. (NC) negative control group; (Bov) mice treated with bovicin HC5; (PC) positive control group.

No β-lactoglobulin (β-LG) was detected in serum samples obtained before β-LG administration or in samples from the NC group after administration of β-LG. In sera obtained from animals of the PC group, significant amounts of β-LG were detected after 0.5, 1 and 2 h of β-LG administration (3.47 mg ml^-1^, 3.53 mg ml^-1^ and 12.14 mg ml^-1^, respectively). After 5 h of administration, β-LG could not be detected in the PC group, suggesting that β-LG clearance required at least 5 h to occur. In the Bov group, low concentrations of β-LG (1.08 mg ml^-1^) were detected in animal sera after 5 h of β-LG administration (Figure 2).

### Oral administration of bovicin HC5 and ovalbumin induce histological and morphometric alterations in the intestine of BALB/c mice

**Figure 3:**
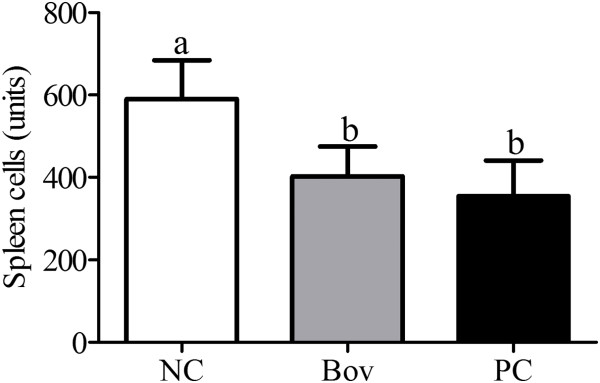
**Comparison of the total number of splenocytes among experimental groups.** Data are shown as average ± SD, from two independent experiments (N = 8 mice per group). Statistically significant differences among treatments by the Dunn’s multiple comparison test (p < 0.05) were indicated by different lowercase letters (“a” or “b”) above the error bars. (NC) negative control group; (Bov) mice treated with bovicin HC5; (PC) positive control group.

No alterations were identified in the liver and heart of animals from all the groups analyzed (data not shown). A significant decrease in the total number of spleen cells was observed in Bov and PC groups, when compared to the NC group (Figure 3).

**Figure 4:**
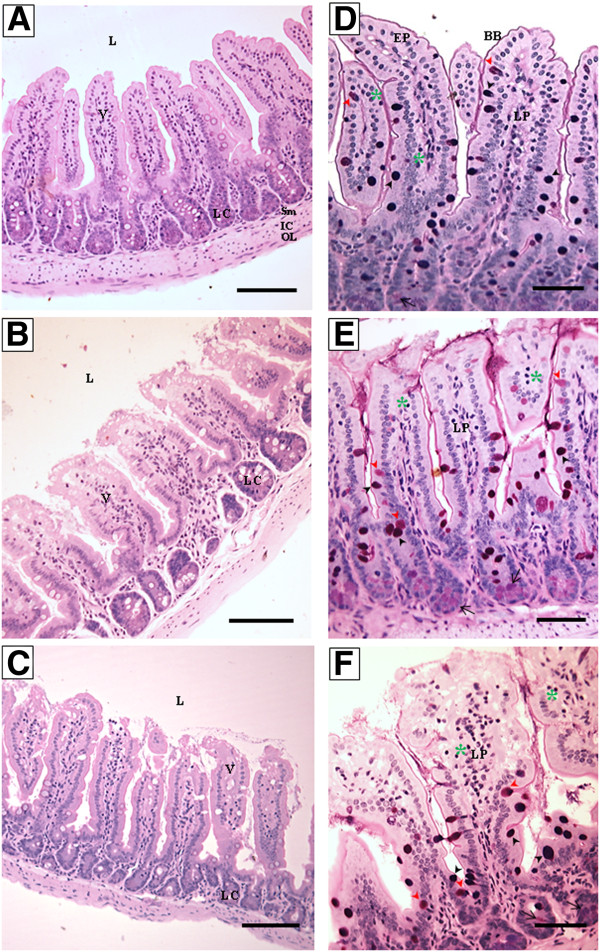
**Photomicrographs of longitudinal sections of small intestine of the experimental groups.** Jejunum segments were collected and processed for optical microscopy analysis at the end of the experiment (day 58) (N = 8 mice per group). (NC), negative control group, figures **A** and **D**; (Bov) mice treated with bovicin HC5, figures **B** and **E**; (PC) positive control group, figures **C** and **F**. The sections were stained with hematoxylin and eosin (HE; left panel) or PAS/Alcian Blue (right panel). Abbreviations: L: lumen; EP: simple cuboidal epithelium; BB: brush border; V: villum; LP: lamina propria; LC: Lieberkühn crypt; Sm: submucosa; IC: inner circular muscle layer; OL: outer longitudinal muscle layer. The asterisks indicate intraepithelial lymphocytes; simple arrow indicates Paneth cells. Black arrow head indicates goblet cells PAS/AB^+^; red arrow head indicates PAS^+^ cells. Right panel – Scale bar: 100 μm; Left panel – Scale bar: 50 μm.

The small intestine of the NC group presented a well-preserved villi and crypts, with intact intestinal layers (Figure 4A and 4D). In the Bov group, the severity of the effects varied among the animals and major alterations were observed in the lamina propria (mild edema) and in the apical portion of the villi, with a “worst case scenario” being presented in Figure 4B and 4E. As expected, the animals from the PC group developed intestinal inflammation, characterized by inflammatory cell infiltration, tissue destruction, epithelial exulceration, edema and congestion of the lamina propria (Figure 4C and 4F).

Morphometric analysis of the small and large intestine of the animals treated with bovicin HC5 or ovalbumin showed some impairment of the intestinal structure integrity, but the severity of the alterations caused by bovicin HC5 and ovalbumin was clearly different.

**Figure 5:**
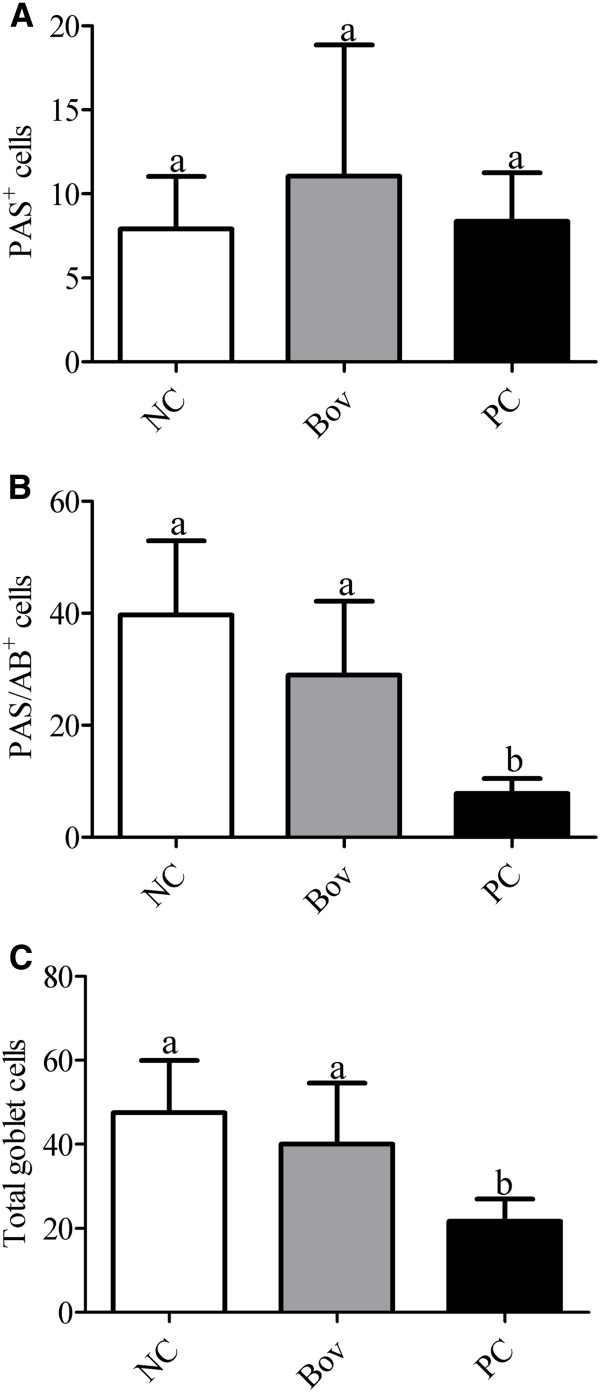
**Comparison of the mucopolysaccharides production and number of total goblet cells among experimental groups. (A)** PAS^+^ cells; **(B)** PAS/AB^+^ cells; **(C)** Total number of goblet cells. Data are shown as average ± SD, from two independent experiments (N = 8 mice per group). Statistically significant differences among treatments by the Dunn’s multiple comparison test (p < 0.05) were indicated by different lowercase letters (“a” or “b”) above the error bars. (NC) negative control group; (Bov) mice treated with bovicin HC5; (PC) positive control group.

The number of PAS^+^ cells, which secrete only neutral mucopolysaccharides, did not differ among the groups (Figure 5A), and cells secreting exclusively acid mucins (AB^+^ cells) were not detected. The majority of goblet cells in NC group was PAS/AB^+^ cells, which secrete both neutral and acidic mucopolysaccharides (83% of the total number of goblet cells). The number of PAS/AB^+^ cells did not differ between the NC and Bov groups, but it was significantly reduced in PC group (p < 0.05, Figure 5B). No differences were observed in the total number of goblet cells in the small intestine of Bov group, when compared to the NC group. However, the total number of goblet cells in the small intestine of PC group was reduced when compared to Bov and NC groups (p < 0.05, Figure 5C).

**Figure 6:**
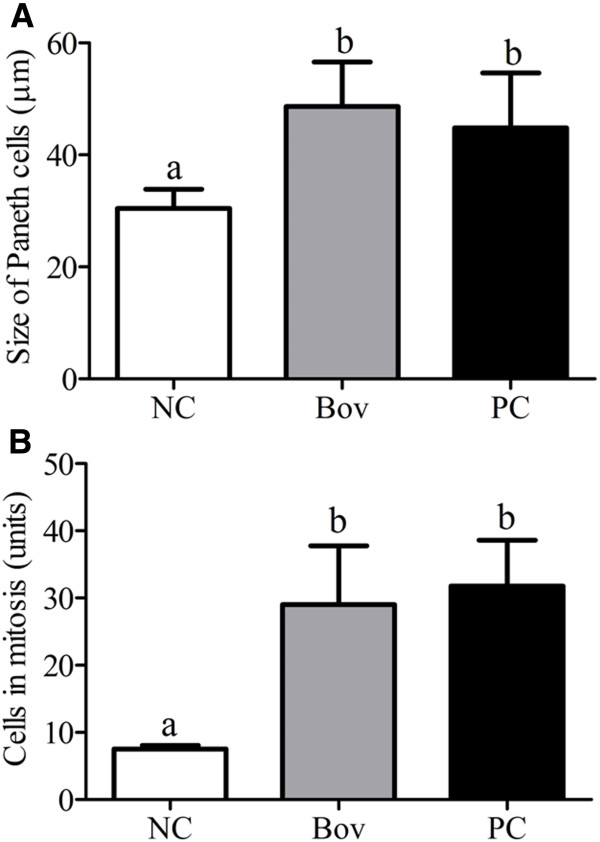
**Analysis of the Lieberkuhn glands.** Size of Paneth cells (**A**) and number of cells in mitosis (**B**) at the small intestinal crypts of the experimental groups. Data are shown as average ± SD, from two independent experiments (N = 8 mice per group). Statistically significant differences among treatments by the Dunn’s multiple comparison test (p < 0.05) were indicated by different lowercase letters (“a” or “b”) above the error bars. (NC) negative control group; (Bov) mice treated with bovicin HC5; (PC) positive control group.

**Figure 7:**
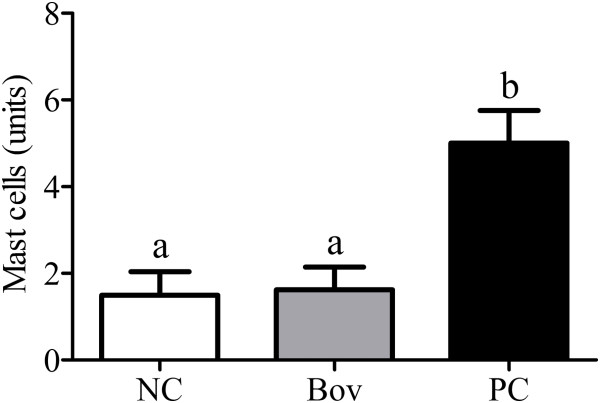
**Number of mast cells in small intestine of the experimental groups.** Longitudinal sections from jejunum segments were stained with toluidine blue/sodium borate (1%), and the mast cells were counted in the mucosa and submucosa. Data are shown as average ± SD, from two independent experiments (N = 8 mice per group). Statistically significant differences among treatments by the Dunn’s multiple comparison test (p < 0.05) were indicated by different lowercase letters (“a” or “b”) above the error bars. (NC) negative control group; (Bov) mice treated with bovicin HC5; (PC) positive control group.

Analysis of the Lieberkühn glands indicated hypertrophy of Paneth cells (Figure 6A) and an increase in the number of mitotic cells (Figure 6B) in Bov and PC groups when compared to the NC group (p < 0.05), although no differences were observed between Bov and PC groups (p > 0.05). No alteration on the number of mast cells on jejunum segments (mucosa and submucosa) was observed between Bov and NC groups, although a significant increase has been observed in PC group (p < 0.05) (Figure 7).

In PC group, the jejunum segments demonstrated a significant increase (p < 0.05) in the number of mast cells from the mucosa and submucosa (Figure 7), when compared to Bov and NC groups.

**Figure 8:**
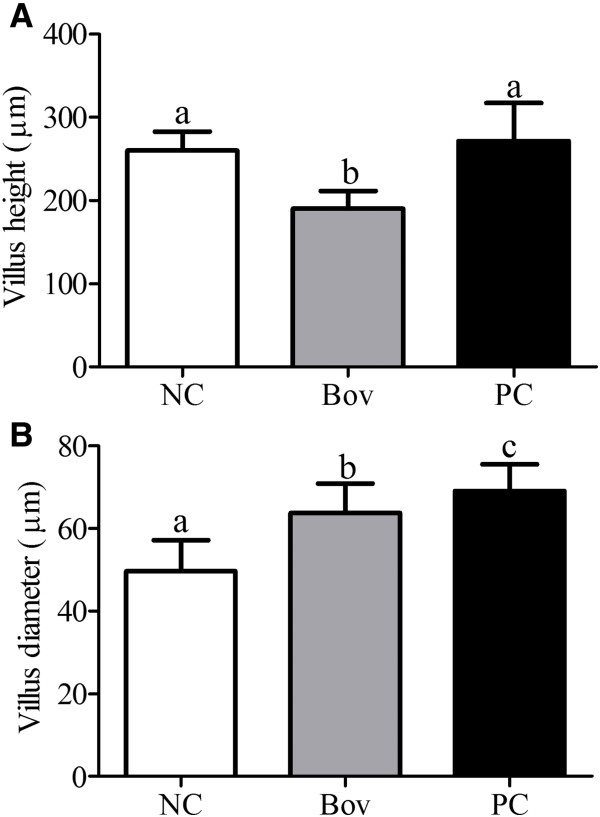
**Morphometric analysis of the small intestinal villi.** Panel (**A**) and panel (**B**) show the height and diameter of the small intestinal villi, respectively. Data were shown as average ± SD, from two independent experiments (N = 8 mice per group). Statistically significant differences among treatments by the Dunn’s multiple comparison test (p < 0.05) were indicated by different lowercase letters (“a”, “b” or “c”) above the error bars. (NC) negative control group; (Bov) mice treated with bovicin HC5; (PC) positive control group.

In the small intestine of animals from the Bov group, significant villous atrophy accompanied by villi enlargement was observed. In PC group, the increase of the villous diameter was even more pronounced when compared to the Bov group (p < 0.05), although the height of the villi was not altered, when compared to NC group (Figure 8).

**Figure 9:**
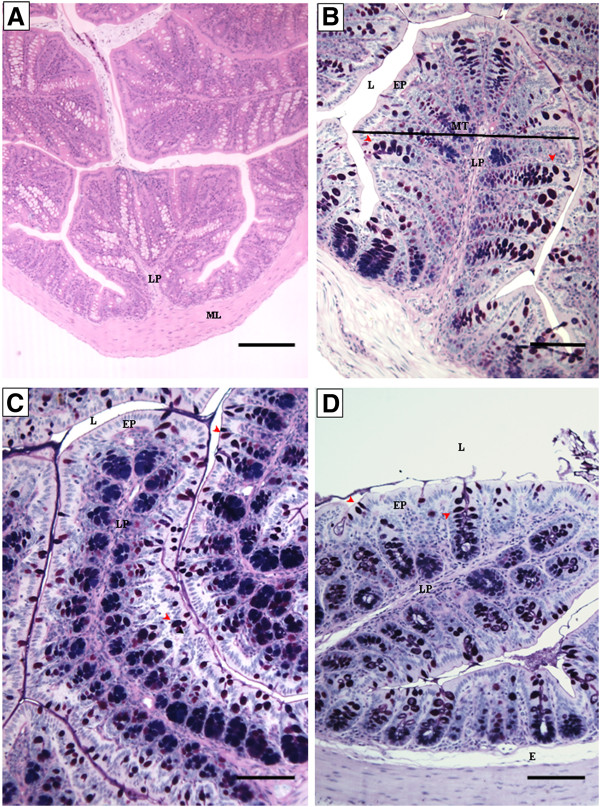
**Photomicrographs of longitudinal sections of large intestine of the experimental groups.** Large intestine segments were collected and processed for optical microscopy analysis at the end of the experiment (day 58) (N = 8 mice per group). (NC), negative control group, figures **A** and **B**; (Bov) mice treated with bovicin HC5, figure **C**; (PC) positive control group, figure **D**. The sections were stained with hematoxylin and eosin (HE; figure **A**) or PAS/Alcian Blue (figures **B-D**). Abbreviations: EP: simple cuboidal epithelium; LP: lamina propria; MT: mucosal thickness; E: edema; ML: muscle layer. Red arrow head indicates goblet cells. Scale bar = 200 (figure A) or 100 μm (figures **B, C** and **D**).

**Figure 10:**
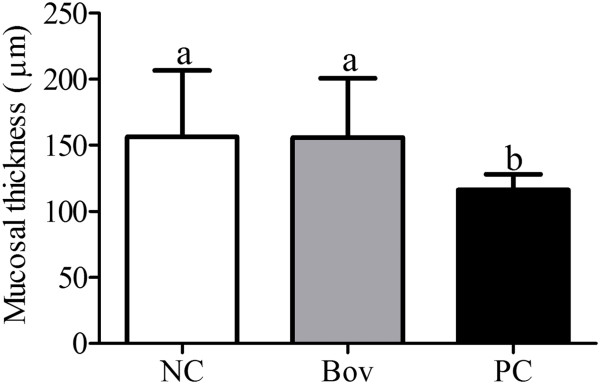
**Comparison of the mucosal thickness of the large intestine among the experimental groups.** Data are shown as average ± SD, from two independent experiments (N = 8 mice per group). Statistically significant differences among treatments by the Dunn’s multiple comparison test (p < 0.05) were indicated by different lowercase letters (“a” or “b”) above the error bars. (NC) negative control group; (Bov) mice treated with bovicin HC5; (PC) positive control group.

The large intestine of the NC group was normal and with a homogenous aspect (Figure 9A and 9B). The effects of bovicin HC5 and ovalbumin were less evident in the large intestine of the animals. No differences on epithelium structure or cellularity were detected in Bov group (Figure 9C), while a moderate edema at the lamina propria (Figure 9D) and a significant reduction at the mucosal thickness (Figure 10) were detected among the animals from the PC group (p < 0.05).

### Assessment of the immunostimulatory effects on spleen and small intestine of animals treated with bovicin HC5 or ovalbumin

**Figure 11:**
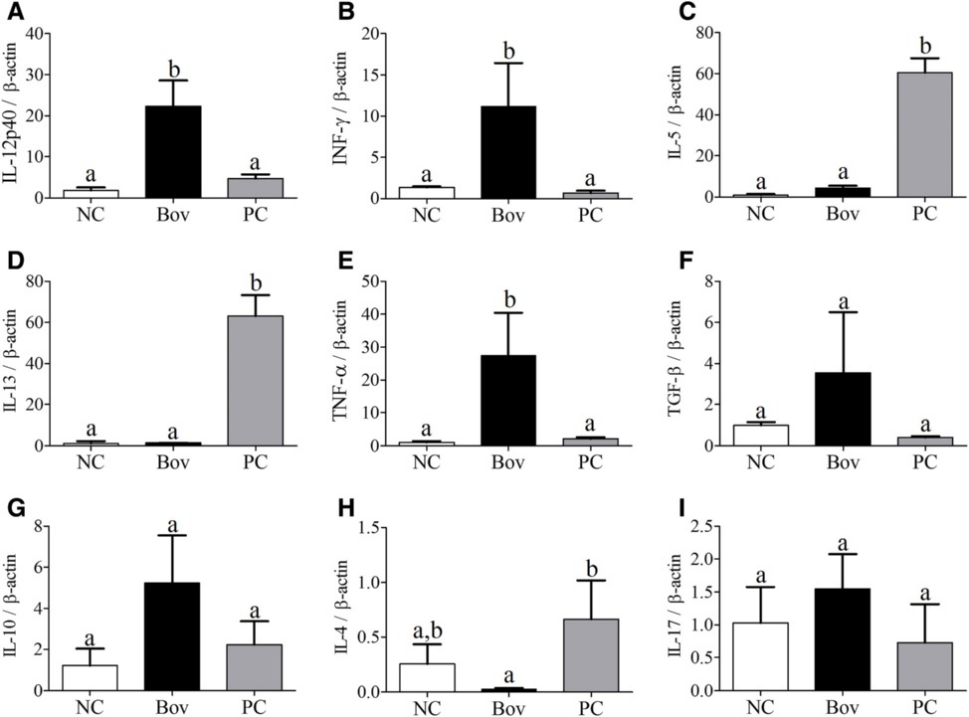
**Cytokine production in small intestine of five-week old female BALB/c mice treated with bovicin HC5 or ovalbumin.** The relative expression of IL-12p40 **(A)**, IFN-γ **(B)**, IL-5 **(C)**, IL-13 **(D)**, TNF-α **(E)**, TGF-β **(F)**, IL-10 **(G)**, IL-4 **(H)** and IL-17 **(I)** mRNA was determined by real time-PCR and calculated by reference to the β-actin in each sample, using the threshold cycle (Ct) method. Results are shown as the mean value ± SD of duplicate samples from three independent mice within the NC, Bov and PC groups. Differences among treatments were indicated by different lowercase letters and were considered statistically significant by the Bonferroni multiple comparison test (p < 0.05). (NC) negative control group; (Bov) mice treated with bovicin HC5; (PC) positive control group.

There was no difference in relative gene expression of cytokines in the spleen when the means of the Bov and NC groups were compared. Only the IL-13 mRNA expression differed among the groups, with the PC group showing the highest expression levels in the spleen (p < 0.05) (Additional file 1). In the small intestine, the relative expression of IL-12, INF-γ and TNF-α was significantly higher for the Bov group (p < 0.05, Figure 11A, 11B and 11E), while the IL-5, IL-13 and IL-4 mRNA expression was significantly higher in the PC group (p < 0.05, Figure 11C, 11D and 11H). The mRNA levels of TGF-β, IL-10 and IL-17 did not differ between the groups (Figure 11F, 11G and 11I).

## Discussion

In this study, we used a murine model of food-induced enteropathy in order to compare the morphological and immunostimulatory effects of the orally administered bovicin HC5. In our positive control group, the breakdown of mucosal tolerance was obtained by oral administration of the non-tolerogenic antigen ovalbumin (OVA). OVA has become a reference protein for immunological and biochemical studies, being widely used as an antigen for studying allergic diseases in mice [17].

The model used to induce food enteropathy worked properly, and an inflammatory reaction was developed in the small intestine. OVA administration altered the small intestinal architecture, increased protein permeability, caused edema and decrease the mucosal thickness in the large intestine. In contrast, upon oral administration of bovicin HC5, only minor histological alterations indicative of inflammation or alterations on permeability were observed, although an atrophy of the villi and destruction of the apical portion of the villi were detected in some regions of the small intestine.

The degree of impairment of the small intestine could explain the differences observed in weight gain between Bov and PC groups throughout the experiment, since these alterations may have influenced the absorption of nutrients. Saldanha *et al.*[18] reported that BALB/c mice previously sensitized lost weight after challenging with OVA, which remained until the end of the experiment.

In normal conditions, the number of mast cells in the intestine is relatively constant, but hyperplasia can be observed during inflammatory reactions or during stages of remodeling/repair of inflammatory disorders [19]. As a result of the food enteropathy developed upon administration of OVA, we observed increased number of mast cells in the small intestine of PC group. However, no alterations were observed in the mast cell population from the Bov group.

Bacterial products or cell components may induce metaplasia, proliferation and hypersecretion of goblet cells [20]. In this study, animals treated with OVA showed reduced number of goblet cells in the small intestine, and a reduction in the secretion of acidic and neutral mucins. In contrast, the administration of bovicin HC5 did not alter the total number or the pattern of goblet cell secretion. The mucus protects the intestinal wall by limiting the absorption of antigens, and therefore, the hypersecretion of mucopolysaccharides was expected at the PC group, as a characteristic of allergic inflammation and as a result of increased IL-13 expression [21]; therefore, the reduction in the number of cells responsible for mucus secretion observed in PC group may not be related to the reduction in the secretion process per se, but to the limited count fields resulting from the destruction of the villi observed in PC group.

Similar to goblet cells, Paneth cells also play an important role in host intestinal defense mechanisms, contributing to the maintenance of the gastrointestinal barrier by secreting antimicrobial peptides and other compounds in response to bacteria and bacterial antigens [22,23]. The presence of antigens in the gastrointestinal tract also influence the expression and activity of key proteins involved in the regulation of cell proliferation [24]. Hypertrophy of Paneth cells and increased mitotic activity were observed in Bov and PC groups, indicating that despite the loss of villi architecture, secretion of antimicrobial compounds and tissue repair systems remained active, probably as a response to the injuries caused by bovicin HC5 and OVA in the small intestine.

Our results indicate that the effects of bovicin HC5 and ovalbumin administration are more pronounced in the intestine, which can explain the significant reduction in spleen cellularity observed in Bov and PC groups: immune cells probably migrated from the spleen to the intestine, where the main effects were observed.

OVA administration modulated the gut mucosal immunity in BALB/c mice towards significant T_H_2-polarized response, increasing the relative expression of IL-4, IL-5 and IL-13 mRNA. Goya *et al.*[25] also observed increased mRNA levels of the T_H_2 cytokines IL-4, IL-5 and IL-13, as well as a decrease of INF-γ expression in the lungs of OVA-treated mice. A down modulation of regulatory mechanisms, with reduction of TGF-β and IL-10 expression, may be involved in the development of food allergy [26], but this pattern of cytokine expression, although expected, was not observed in this study.

The modulation of the host immune system induced by bacteriocins is a phenomenon much less understood when compared to other peptides or proteins, such as proteins extracted from mushrooms (such as LZ-8 (13 kDa) [27], Fip-vvo (15 kDa) [28] and FIP-fve (114 aa) [29]) and host-defense peptides [30,31].

In contrast to the T_H_2-polarized response elicited by OVA, higher mRNA expression for the T_H_1 cytokines TNF-α, IL-12 and INF-γ were observed in the intestine of bovicin HC5-fed mice. Liu *et al. *[32] also demonstrated significant induction of IFN-γ after administration of the yam tuber storage protein dioscorin. Human cathelicidin LL-37 modulated the activity of IFN-γ on a variety of cell types [33], and pre-treatment with LL-37 induced IFN-γ production by monocytes, enhancing monocyte-derived dendritic cell functions, such as IL-12 secretion and T_H_1-polarized co-stimulatory activity [34].

## Conclusions

In the present work, for the first time, the effects of the oral administration of bovicin HC5 to an animal model were described. The bovicin HC5 concentration administrated to the animals (micromolar range) was greater than the quantities required for *in vitro* antimicrobial activity (nanomolar range). We have previously demonstrated that bovicin HC5, in higher con-centrations, was able to permeabilize membranes in an unspecific way [13], but one should bear in mind that antimicrobial peptides can also modulate the microbial community composition in the intestine which could explain the partial destruction of small intestine cells caused by bovicin HC5 administration. Nonetheless, the impairment of the intestinal cells induced by bovicin HC5 neither altered the gut permeability nor was typical of an enteropathy process. Regarding the immunostimulatory effects, the results confirmed that bovicin HC5 was able to stimulate the immune system of BALB/c mice at local level (gut immune system), by influencing the cytokine release towards T_H_1-polarized response.

Proper pharmacokinetic studies will be needed to determine if bovicin HC5 can resist passage through the adverse conditions in the GI tract (low pH, presence of peptidolytic and proteolytic enzymes), but it should be noted that animals treated with bovicin HC5 showed more pronounced effects in the intestine compared to the animals in the negative control groups. These results suggest that the oral administration of bovicin HC5 might be a promising strategy to control microbial infections, manipulate microbial community composition or modulate immunological responses in the GI tract of the host animal.

## Methods

### *Streptococcus bovis* HC5 and bovicin HC5

*Streptococcus bovis* HC5 growth and bovicin HC5 extraction were performed as previously described [11]. Purification of bovicin HC5 was performed by reversed phase-HPLC [13] and the purity of bovicin HC5 was confirmed by electrospray mass spectrometry to be always greater than 95%.

Bovicin HC5 stock solutions (1 mg ml^-1^ in PBS (10 mM, pH 7.2)) were stored at −20°C until use. Protein concentration was determined using a bicinchoninic acid protein assay (Pierce Chemical Corp., Bonn, Germany), with bovine serum albumin as the standard.

### Experimental animals

The BALB/c mice used in this study were housed in an animal facility at the Universidade Federal de Viçosa, according to standards and guidelines as set forth in the Animal Welfare Legislation, the Guide for the Care and Use of Laboratory Animals, the Association for the Assessment and Accreditation of Laboratory Animal Care (AAALAC) and the National Council for Animal Experimentation Control (CONCEA), following approval by the Institutional Animal Care and Use Committee (IACUC) of the Universidade Federal de Viçosa under the protocol number CEUA/UFV 97/2011.

Five-week-old female BALB/c mice were randomly divided into three experimental groups: Group 1, untreated mice (negative control, NC group); Group 2, mice given purified bovicin HC5 (Bov group); Group 3, mice given ovalbumin (Sigma Chemicals Co., St. Louis, MO, 99% of purity) (positive control, PC group). Two independent experiments were performed and a total number of eight animals were used per experimental group.

The sensitization procedure was developed based on previously established protocols [18]. Mice from the Bov group were subcutaneously sensitized with bovicin HC5 (4 μg/g animal weight/day or approximately 70 μg/animal [35]), while animals from the PC group were sensitized with ovalbumin (100 μl of a 1 mg ml^-1^ stock solution in sterile ultrapure water, or 100 μg OVA/animal). Aluminum hydroxide was used as adjuvant (50 μl; 20 mg ml^-1^ stock solution in sterile saline) at the first sensitization (day 0). After three weeks, each mouse group was subcutaneously boosted (without the use of adjuvant) with the respective substances (second sensitization, day 21). The NC group was sensitized with sterile PBS (10 mM, pH 7.2), using the same procedure described above.

PBS, bovicin HC5 or ovalbumin (100 μl) were administered without adjuvant to the NC, Bov and PC groups, respectively, by daily gavages (*18-gauge* stainless steel feeding needles). Oral administration started one week after the second sensitization (day 28) and continued for 30 days uninterruptedly (day 58). The mice were weekly weighted and behavior, general appearance and adverse reactions were monitored daily.

### Gut permeability

The gut permeability was determined by the uptake of β-lactoglobulin (β-LG) following challenge ([36], with modifications]). At the end of the trial period (day 58), the animals of the NC, Bov and PC groups, were orally challenged with 200 μl of the respective samples (PBS, bovicin HC5 or ovalbumin). After thirty minutes, the animals received an oral dose of β-LG (200 μl; 100 mg ml^-1^ solution in distilled water) (Sigma Chemicals Co., St. Louis, MO, 90% of purity).

Blood samples were collected from the orbital plexus under light isoflurane anesthesia, after 0.5, 1, 2 and 5 h of the β-LG administration. The samples were kept at room temperature for 2 hours, and the sera were centrifuged (Eppendorf®, Centrifuge 5415C, Hamburg, Germany) at 12,000 × g, 5 min, room temperature. Sera were used for the quantification of β-LG by FPLC, using a cationic change column (Mono Q HR 5/5). The column was equilibrated with buffer A (20 mM Tris) and the β-LG was eluted with a linear gradient of 25 to 50% buffer B (20 mM Tris, 1 M NaCl), 22°C, and flow rate of 1 ml min^-1^. Absorbance was monitored at 220 and 280 nm.

The concentration of β-LG in animal sera was determined using a calibration curve with known concentrations of β-LG (0; 6.25; 12.5; 25.0; 50.0 mg ml^-1^) mixed to pre-immune serum of the animals from each group. The pre-immune serum corresponded to the sera collected prior to the initial sensitization procedure. Serum samples before β-LG administration were used as negative control. All analyses were performed in duplicate.

### Histological and morphometric analysis

On day 58 the heart, liver, spleen and gut of the all the mice were aseptically collected, washed in PBS buffer (10 mM, pH 7.2), fixed in Carson formalin solution [37], dehydrated and embedded in resin (Historesin®, Leica). Transverse and longitudinal, 3 μm thick tissue sections were obtained and stained with hematoxylin and eosin (H&E), toluidine blue/sodium borate (1%) or with Alcian Blue (pH 2.5) combined with periodic acid-Schiff (PAS) [38], depending on the histological analysis that would be performed.

Ten fields of longitudinal sections stained with H&E were randomly selected and visualized with a 10× objective lens in order to perform the morphological analysis of the organs selected (villi height and width were determined from an area of 17 mm^2^ per animal; for mucosal thickness, an average of twenty measurements were obtained from each animal). The spleen cells were counted using ten fields of longitudinal sections visualized with a 40× objective lens, in an area of 0.23 mm^2^ per animal. For quantitative and qualitative analysis of goblet cells, ten fields of longitudinal sections (area of 1 mm^2^) stained with Alcian Blue-PAS were randomly selected and visualized with a 20× objective lens; the mucins produced by goblet cells were identified by differential staining (acid mucins in blue, neutral mucins in red, and mixed acid and neutral mucins in purple). The mast cells were counted using ten longitudinal sections stained with toluidine blue/sodium borate (1%) and visualized with a 40× objective lens; an area equivalent to 20 jejunum villi (mucosa and submucosa) was evaluated for each animal.

Digital images were captured with a light microscope (Olympus AX 60), coupled to a digital camera (Q-Color 3, Olympus). The morphometric analyzes were performed with the image analysis program Image Pro Plus 4.0 for Windows (Media Cybernetics). The results were shown as mean values ± standard error of the mean.

### Analysis of relative gene expression by real-time PCR

The whole spleen and jejunum segments (100 mg of tissue) were aseptically removed, washed in sterile PBS (10 mM, pH 7.2), and individually manipulated. Cells obtained from the spleen were washed with saline (0.85%), centrifuged at 7,500 × *g* for 5 min (Eppendorf®, Centrifuge 5415C, Hamburg, Germany), at room temperature, and the erythrocytes were lysed using a hemolytic solution (155 mM NH_4_Cl, 10 mM KHCO_3_, pH 7.2). The splenocytes were centrifuged again and the supernatant was discarded. A jejunum segment of 6 cm was removed and washed three times with saline (0.85%), for removal of waste.

The splenocytes and jejunum segments were homogenized in Tri Reagent (Sigma®) to isolate total RNA, following the manufacturer’s instructions. RNA yield was analyzed spectrophotometically at 260 nm (Ultraspec 3000, Pharmacia Biotech, Piscataway, NJ). From each group (NC, Bov and PC), three samples from different animals showing highest RNA yield were chosen for cDNA synthesis. Complementary DNA (cDNA) was synthesized through a RT reaction (M-MuLV reverse transcriptase, Promega), according to the manufacturer’s instructions. Real-time RT-PCR was conducted with a final volume of 25 μL containing 2.5 ng of cDNA, SYBR-green PCR Master Mix (Applied Biosystems, Warrington, UK), oligo(dT) cDNA as the PCR template, and 450 nM specific primers. Real-time RT-PCR was performed on the Gene Amp®5700 Sequence Detection System Version 1.3 (Applied Biosystems) and the cycling parameters used were 95°C for 10 min, 40 cycles at 94°C for 1 min, 56°C for 1 min, and 72°C for 2 min, followed by the standard denaturation curve. The sequences of murine primers used in this study (Table 1) were previously described [9].

**Table 1:** **Sequences of sense (S) and antisense (AS) *primers *used for real time-RT-PCR analysis (18)**

***Primers***	**Sequences**
β-actin S	5^′^ AGC TGC GTT TTA CAC CCT TT 3^′^
β-actin AS	5^′^ AAG CCA TGC CAA TGT TGT CT 3^′^
IL-10 S	5^′^ TGG ACA ACA TAC TGC TAA CC 3^′^
IL-10 AS	5^′^ GGA TCA TTT CCG ATA AGG CT 3^′^
IL-4 S	5^′^ CTG ACG GCA CAG AGC TAT TGA 3^′^
IL-4 AS	5^′^ TAT GCG AAG CAC CTT GGA AGC 3^′^
IL-5 S	5^′^ GAG GTT ACA GAC ATG CAC CAT T 3^′^
IL-5 AS	5^′^ TCA GTT GGT AAC ATG CAC AAA G 3^′^
IL-13 S	5^′^ ACC AAC ATC TCC AAT TGC AA 3^′^
IL-13 AS	5^′^ ATG CAA TAT CCT CTG GGT CC 3^′^
TNF-α S	5^′^ TGT GCT CAG AGC TTT CAA CAA 3^′^
TNF-α AS	5^′^ CTT GAT GGT GGT GCA TGA GA 3^′^
IL-12 p40 S	5^′^ AGC ACC AGC TTC TTC ATC AGG 3^′^
IL-12 p40 AS	5^′^ GCG CTG GAT TCG AAC AAA G 3^′^
IFN-γ S	5^′^ GCA TCT TGG CTT TGC AGC T 3^′^
IFN-γ AS	5^′^ CCT TTT TCG CCT TGC TGT TG 3^′^
TGF-β S	5^′^ GCT GAA CCA AGG AGA CGG AAT 3^′^
TGF-β AS	5^′^ GCT GAT CCC GTT GAT TTC CA 3^′^
IL-17 S	5^′^ GCT CCA GAA GGC CCT CAG A 3^′^
IL-17 AS	5^′^ CTT TCC CTC CGC ATT GAC A 3^′^

The results were demonstrated as relative level of gene expression in the experimental group, by reference to the β-actin gene in each sample, using the cycle threshold (Ct) method. Measurements were conducted in duplicates and the fold increase expression was calculated by using the expression 2^DCt, according to the instructions from Applied Biosystems User’s Bulletin #2 (P/N 4303859). Results were shown as mean values ± standard deviation.

### Statistical analysis

The means of the groups were evaluated by analysis of variance (ANOVA) followed by the Dunn’s or Bonferroni’s post test. A probability value of less than 0.05 was considered statistically significant, and all the comparisons were performed using the GraphPad Prism 5.00 software (GraphPad Software, San Diego California, USA).

## Competing interests

The authors have declared no competing interests.

## Authors’ contributions

ADP performed all the experiments. KMF carried out the histological analysis. RSD and ASR participated in the collection of immunological data. LLO, CAN and SOP participated in the analysis and interpretation of data. ADP, HCM and SOP participated in the design of the study. ADP and HCM prepared the manuscript. All authors read and approved the final manuscript.

## Supplementary Material

Additional file 1 **Cytokine production in spleen of five-week old female BALB/c mice treated with bovicin HC5 or ovalbumin.** The relative expression of IL-12p40 (A), IFN-γ (B), IL-5 (C), IL-13 (D), TNF-α (E), TGF-β (F), IL-10 (G), IL-4 (H), IL-17 (I) mRNA was determined by real time-PCR and calculated by reference to the β-actin in each sample, using the threshold cycle (Ct) method. Results are shown as the mean value ± SD of duplicate samples from three independent mice within the NC, Bov and PC groups. Differences among treatments were indicated by different lowercase letters and were considered statistically significant by the Bonferroni multiple comparison test (p < 0.05). (NC) negative control group; (Bov) mice treated with bovicin HC5; (PC) positive control group. (TIFF 10328 kb) Click here for file
